# Rapid, Affordable and Portable Medium-Throughput Molecular Device for Zika Virus

**DOI:** 10.1038/srep38223

**Published:** 2016-12-09

**Authors:** Kamfai Chan, Scott C. Weaver, Pui-Yan Wong, Sherly Lie, Eryu Wang, Mathilde Guerbois, Siva Praneeth Vayugundla, Season Wong

**Affiliations:** 1 AI Biosciences, Inc., College Station, TX, 77845, USA; 2 Institute for Human Infections and Immunity and Departments of Microbiology & Immunology and Pathology, University of Texas Medical Branch, Galveston, TX, 77555, USA

## Abstract

Zika virus (ZIKV) has gained global attention as an etiologic agent of fetal microcephaly and Guillain-Barré syndrome. Existing immuno-based rapid tests often fail to distinguish between Zika and related flaviviruses that are common in affected regions of Central and South Americas and the Caribbean. The US CDC and qualified state health department laboratories can perform the reverse transcription polymerase chain reaction (RT-PCR) ZIKV test using highly sophisticated instruments with long turnaround times. The preliminary results of a portable and low-cost molecular diagnostics system for ZIKV infection are reported here. In less than 15 minutes, this low-cost platform can automatically perform high quality RNA extraction from up to 12 ZIKV-spiked urine samples simultaneously. It can also perform reverse transcription recombinase polymerase amplification reaction (RT-RPA) in ≤15 minutes. The fluorescent signal produced from probe-based RT-RPA or RT-PCR assays can be monitored using LEDs and a smartphone camera. In addition, the RT-RPA and RT-PCR assays do not cross-react with dengue and chikungunya viral RNA. This low-cost system lacks complicated, sensitive and high cost components, making it suitable for resource-limited settings. It has the potential to offer simple sample-to-answer molecular diagnostics and can inform healthcare workers of patients’ diagnosis promptly.

Zika virus (ZIKV) is an emerging mosquito-borne pathogen (family *Flaviviridae*, genus *Flavivirus*) that was first isolated in 1947 from a rhesus monkey in Uganda’s Zika forest^1^. The current hypotheses for transmission postulates that ZIKV was transmitted to humans by infected *Aedes spp*. mosquitoes^2^,^3^. This once obscure pathogen recently became a major concern for global public health authorities after it was connected with fetal microcephaly^4^,^5^ and Guillain-Barré syndrome (GBS)^6^,^7^. The World Health Organization has declared ZIKV a public health emergency and urged fast-tracked development of Zika virus diagnostics. Because of the potential impact to the U.S. population, President Obama has also called for the rapid development of tests, vaccines and treatments to fight ZIKV.

ZIKV infection has been poorly described in the past, as it was previously believed to be a benign, self-limiting illness^8^. Thus, ZIKV infection has probably been underdiagnosed and underreported in endemic settings or in returning travelers^9^. In addition to nonspecific clinical features, a diagnosis of ZIKV is challenging because standard serological approaches, such as IgM antibody detection or seroconversion, are limited in value due to cross-reactivity in patients that have previously been infected by other flaviviruses (including dengue and chikungunya) that circulate in the endemic regions^10^.

ZIKV viremia found in human infection ranges from 10^2^ to 10^6 ^PFU/mL^10^,^11^,^12^,^13^,^14^. The molecular detection of viral RNA in serum using a RT-PCR protocol described by Lanciotti *et al*. during investigations of the ZIKV outbreak on Yap Island has been deemed suitable for acute phase diagnosis of ZIKV^10^,^14^,^15^,^16^,^17^. The specificity of this protocol for ZIKV amplification has been confirmed against other arboviruses, especially dengue, which was co-circulating in French Polynesia during the ZIKV outbreak. In addition to serum, the diagnostic utility of urine and saliva as sources for detection of ZIKV RNA by real-time RT-PCR has also been reported^18^,^19^. The use of urine as a diagnostic specimen has the advantages of noninvasive collection and ample volume of sample in collections. The recent paper by Gourinat *et al*. shows evidence of virus secretion in urine for more than 10 days after the onset of disease and detection of ZIKV RNA with an estimated maximum viral load of 0.7–2.2 × 10^6 ^copies/mL^18^. Others also reported prolonged detection of ZIKV RNA in urine during the epidemic in Brazil^20^. These recent studies are in agreement with previous reports that other *flavivirus* genomes, such as those of dengue^21^, West Nile^22^ and most recently ZIKV RNA^23^ can be detected in urine longer than in serum. These reports suggest that urine samples are a superior choice for diagnosis of ZIKV infection due to the higher RNA load and longer duration compared to serum. For these reasons, urine was chosen in this study as the sample specimen to demonstrate the diagnostic utility of the novel platform for detection of ZIKV RNA using isothermal amplification techniques such as real-time reverse transcription recombinase polymerase amplification (RT-RPA) and the more traditional real-time RT-PCR.

While PCR and RT-PCR are considered the gold standard in molecular detection of pathogens, isothermal amplification techniques are being sought as alternatives, because they do not require thermal cycling. Among existing isothermal techniques, RPA operates optimally between 39 and 42 °C^24^. Unlike PCR, RPA does not require an initial heat denaturation step to unwind double-stranded DNA. The primer–recombinase complex, along with single-strand binding proteins (SSBs), ensure the unwinding stability of nucleic acid during the various exchange processes^25^. The addition of exonuclease III also allows the use of a fluorogenic exo probe for real-time fluorescence detection. RPA reactions commercialized by TwistDx (Cambridge, UK) can give positive results in 5 to 10 min. The progress of amplification reactions using an exo-probe can be monitored with a lab-based fluorescence detector^26^,^27^,^28^. A low-cost approach using blue LEDs as excitation light source and a smartphone camera with a colored filter can also be used to capture fluorescent emission signals^28^,^29^. For the detection of RNA, reverse-transcriptase is added so “one-step” RT-RPA can be used^30^,^31^,^32^,^33^,^34^,^35^.

To address the current Zika virus outbreak, a portable and reliable molecular system using well–established methods offers the most likely solution to the critical challenges impeding diagnosis. Accurate diagnosis requires reproducible and efficient nucleic acid extraction and purification steps prior to the implementation of nucleic acid-based detection methods, such as PCR and some isothermal nucleic acid amplification methods^10^,^20^,^36^,^37^. However, these techniques are difficult to perform outside of laboratory settings, require technical expertise to perform, and utilize equipment that is incompatible with or too costly for use in low-resource locations. While a few automated point-of-care (POC) molecular systems, such as FilmArray (bioMérieux/BioFire Diagnostics, Salt Lake City, UT) and GeneXpert Omni (Cepheid, Sunnyvale, CA) systems, are available commercially for a few infectious diseases, they are too expensive to be deployed to most ZIKV-affected regions, and each device can only handle one sample at a time. To meet the throughput requirements for the ongoing ZIKV epidemic, multiple units of these platforms will likely be required at a single location, which drastically increases the hardware cost for implementing POC testing. A recent publication mentioned the novel use of a toehold switch sensor to detect ZIKV on a paper substrate, but it is still in the early stages of development and will not likely be optimized soon enough for the current threat of ZIKV^38^.

To meet the immediate challenge of ZIKV testing and surveillance, a low-cost, robust diagnostic platform for molecular detection of ZIKV in low-resource settings was developed. The system addresses the limitations preventing the practical deployment of nucleic acid-based molecular diagnostics platforms by combining automated magnetic particle (MP)-based nucleic acid extraction with rapid real-time RT-RPA or RT-PCR and post-amplification fluorescence detection. The platform presented here is inspired by recent developments in consumer-level 3D printers and printing technology. Figure 1a shows the concept of a low-cost 3D printer converted into a robotic device to carry out high quality, automated DNA/RNA extraction and nucleic acid amplification.

One advantage of the described approach is that the modifications to the 3D printers are completely reversible. A $750 3D printer (Printrbot Simple Metal equipped with heated bed as shown in Fig. 1b) was successfully converted for processing 8 or 12 samples simultaneously in this study. ZIKV spiked urine samples were added to the well containing lysis buffer and NA binding MPs. Automated RNA extraction was performed by the 3D printer, requiring only 15 min for up to 12 samples. The purified RNA was eluted into the elution buffer and a few microliters of the template were used for each real-time RT-RPA reaction. Alternatively, the extracted RNA can be amplified using RT-PCR in the Thermos Thermal Cycler (TTC) that has been developed by the authors. Post RT-PCR viewing of the reaction tubes was achieved by using a smartphone.

The remainder of this manuscript will demonstrate that, using this modified 3D printer, the entire ZIKV detection protocol can be completed in 27–30 min using real-time RT-RPA (15 min extraction and 12 min amplification) or RT-PCR with post amplification inspection (15 min extraction and 15 min RT-PCR). The system will show that the 3D printer can process a wide concentration range of ZIKV inoculated in urine samples. Most importantly, the RT-PCR and RT-RPA assays achieve clinically relevant sensitivity, and the assay does not cross-react with closely related dengue and chikungunya viruses. In short, this proof-of-concept work demonstrates the potential in that rapid and high performing molecular assays can be achieved using very simple instrumentation.

## Methods

### Zika virus and urine specimens and ethics statement

ZIKV (a 2015 strain from Mexico) was harvested in Vero cells 4 days post infection, at a concentration of approximately 2 × 10^7^ plaque-forming units (PFU)/mL. The ZIKV suspension was then mixed with 3 volumes of TRIzol Reagent (Thermo Fisher Scientific [TFS], Waltham, MA), to bring the concentration of ZIKV to 5 × 10^6 ^PFU/mL. Deidentified normal human urine specimen (Catalog# IR100007P) was purchased from Innovative Research (Novi, MI).

### Converting a 3D printer into an automated RNA extraction device

**Figure 1:**
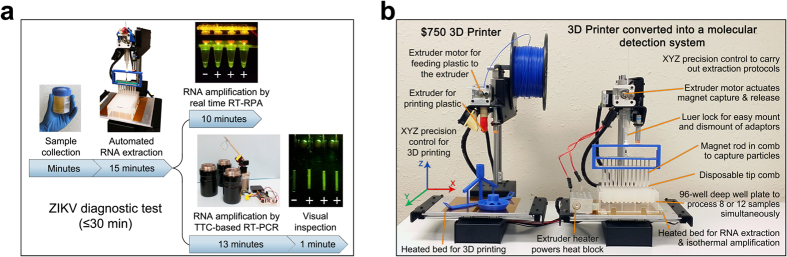
Concept of using modified 3D printer to carry out molecular diagnostics. (**a**) Rapid and low-cost diagnostics for ZIKV using a modified entry-level 3D printer. The printer was reversibly-converted to automate magnetic particle-based nucleic acid extraction. The 3D printer’s motions are controlled by G-codes and can easily be written to perform extraction protocols. A deep-profile 96-well microplate can be used to hold samples and reagents for processing multiple samples. The templates can be amplified by real-time reverse transcription recombinase polymerase amplification reaction (RT-RPA) using the printer’s heating element (a heated bed or filament extruder). Alternatively, the templates can be amplified by RT-PCR using a low-cost Thermos Thermal Cycler. Results were confirmed by post-PCR fluorescence analysis using a smartphone camera. (**b**) Converting a low-cost 3D printer to perform rapid and automated nucleic acid isolation and real-time RPA amplification. The extruder was removed to allow the adaptors to be mounted. The magnetic particle processor attachment (MPPA) was attached to the adaptor by a luer-lock mechanism. Its vertical and lateral movements were controlled by the Z-motor and the X and Y platform control. The disposable tip-comb housed a set of magnetic rods controlled by the extruder motor. The tip-comb and magnetic rods perform MP capture and resuspension via the Z-axis motor and extruder motor. There was no direct contact between the magnets, and the samples and MPs. The printer bed holds a 96-well deep-well microplate or pre-loaded cartridges. Depending on the orientation of the plate, 8 or 12 samples can be processed simultaneously.

Modifications of the 3D printer are shown in Fig. 1b. Previous studies have shown that DNA and RNA can be automatically extracted from clinical samples, using the bioMérieux NucliSENS magnetic extraction reagents and a modified consumer-grade 3D printer (Printrbot Simple, Printrbot, Inc., Lincoln, CA)^39^. The 3D printer weighs 12.4 lb and has overall footprint dimensions of 18″ × 17″ × 13″ (L × W × H). It can be powered by a 12 V (6 amp) computer power supply, which means it also can be powered by a car battery with an inverter. In the initial design, the continuous binding of MPs to the sleeved magnet throughout the washing and elution steps could lead to suboptimal washing of the MPs and/or elution of nucleic acids when RNA (but not DNA) was in the intended target. Therefore, the redesigned magnetic particle processor attachment (MPPA) allows thorough MP resuspension.

In this study, advancements in the manipulation of MPs for more thorough washing were made. Magnetic rods used along with the tip-comb can now be moved in and out of the tip-comb using the extruder motor that was originally designed to move the printing filament into the extruder. This enables capturing and releasing the MPs into different reagents, making the washing steps highly effective. The MPPA consists of a 3D-printed holder attached to a luer-lock syringe through two adaptors. A disposable 8-tip comb (Catalog# 97003500, TFS, Waltham, MA) was securely seated on the holder. A set of 8 axially magnetized neodymium rod magnets (Catalog# D2Y0, K&J Magnetics, Plumsteadville, PA) spaced 9 mm apart were fixed to a 3D-printed plate attached to a metal plate. The metal plate was held by the polylactic acid (PLA) filament with a small disc magnet (Catalog# R125-063, Amazing magnets, LLC, Anaheim, CA) adhered to one end. The vertical movement of the PLA filament and, subsequently, the rod magnets was controlled by the 3D printer’s extruder stepper motor via G-codes.

### RNA extraction from serially diluted ZIKV sample

Dynabeads SILANE Viral NA Kit (TFS) or NucliSENS (bioMérieux) magnetic extraction reagents were aliquoted into a 96-well polypropylene deep well plate (Catalog# 82007-292, VWR). The first two rows were purposely left empty to reconfirm alignment of the plate with the MPPA when the program started. Wash Buffer 1 (500 μL each) was added to each well of the fourth and fifth rows. Wash Buffer 2 (500 μL each) was added to the wells of the sixth and seventh rows. Finally, 100 μL of Elution Buffer or RNA Storage Solution (1 mM sodium citrate, pH 6.4, TFS) was added to each well of the twelfth row. In each well of the third row, 100 μL of ZIKV sample serially diluted (10^−1^, 10^−2^, 10^−3^, 10^−4^, 10^−5^, and 10^−6^) in human urine was mixed with 200 μL of Lysis/Binding Buffer, 75 μL of isopropanol and 20 μL of MP suspension. Sample lysing and nucleic acids binding to MPs were done in the wells at room temperature for 5 minutes. G-code was specially written to instruct the 3D printer to perform the RNA extraction process. Starting with the Lysis/Binding wells, MPs were captured when the rod magnets were inserted into the tip comb and lowered into the wells. MPs were transported from one well to the other by the MPPA, which was precisely controlled by the 3D printer. To disperse the MP pellets for thorough washing or elution, the MPPA was vigorously shaken sideways as the rod magnets were raised. Therefore, RNAs from each sample lysate were bound to the MPs, which were washed twice with Wash Buffer 1 and twice with Wash Buffer 2, and finally eluted in 100 μL of Elution Buffer or RNA Storage Solution. The elution step was carried out at 60 °C to increase efficiency. This heating step was controlled by the heated bed of the 3D printer, and an aluminum block was used to conduct heat from the heated bed to the bottom of the wells of the deep well plate. In the standard protocol, the entire 3D printer-operated extraction process took less than 15 min (5 min lysis and nucleic acids binding, approximately 6 min washing, and 3 min elution).

For comparison, a parallel RNA extraction was performed manually using the QIAamp MinElute Virus Spin kit (Qiagen, Valencia, CA) according to manufacturer’s instructions with minor modifications. Specifically, 100 μL of cell culture-derived ZIKV serially diluted (10^−1^, 10^−2^, 10^−3^, 10^−4^, 10^−5^, and 10^−6^) in human urine was mixed with 100 μL of Buffer AL and 12.5 μL of Qiagen protease solution. After 15 min of 56 °C incubation, 2.5 μL of carrier RNA solution (stock concentration of 1 μg/μL in Buffer AVE) and 125 μL of 95% ethanol were added to the lysate. RNA was extracted from each dilution in duplicate columns, and standard washing steps were performed as recommended by the manufacturer. RNA was eluted with 100 μL of Buffer AVE at room temperature. Of note, the input and output samples’ volume were purposely chosen to be the same in both extraction methods to allow for fair comparison in their efficiency.

### Real-time RT-PCR assay using a commercial thermal cycler

The RNA template extracted by our device was evaluated in a commercial real-time PCR detection system (Bio-Rad CFX-96, Hercules, CA) using a SuperScript III Platinum One-Step Quantitative RT-PCR system (TFS, Waltham, MA). Each 20 μL reaction consisted of 10 μL of the 2× Reaction Mix, 500 nM each of ZIKV 1086 and ZIKV 1162c primers, 250 nM of the FAM-labeled hydrolysis probe (ZIKV 1107-FAM), and 2 μL of the RNA template (see Table 1 for primers and probe sequences). Specificity of the ZIV primers was previously evaluated by testing against dengue, West Nile, yellow fever, and chikungunya viral RNA^10^. The conditions were 50 °C for 5 min (cDNA synthesis), 95 °C for 30 sec, and 45 cycles of 95 °C for 10 sec and 60 °C for 20 sec [or referred to as 300 s/30 s/45 × (10 s/20 s)], with fluorescence detection by a real-time thermal cycler. The duration of each commercial real-time RT-PCR run was approximately 57 min. A much shorter protocol was used when the speed of the commercial thermal cycler was compared to a Thermos Thermal Cycler (TTC) developed by the authors^40^,^41^: 50 °C for 30 sec (cDNA synthesis), 95 °C for 10 sec, and 40 cycles of 95 °C for 5 sec and 60 °C for 10 sec [30 s/10 s/40 × (5 s/10 s)]. The duration of this run was 36 min 47 sec. In most RT-PCR reactions, a no-template control (NTC) and ZIKV negative controls were added in the same run. The negative controls were either template from ZIKV negative urine samples or template containing dengue RNA (DENV-1-4) extracted from the dengue positive control (a mix of heat inactivated DENV-1, -2, -3 and -4 standards) from the CDC’s DENV-1-4 Real Time RT-PCR Assay.

### Rapid detection of ZIKV by RT-RPA assay

For the RT-RPA assay, we designed primers and a TwistAmp exo probe that were modified from the real-time RT-PCR primers and probe for the detection of prM gene of the Zika virus^10^. RPA primers were 30 to 35 bases long as recommended^25^. Therefore, the RPA ZV-F and RPA ZV-R primers have 31 and 30 bases respectively, and the amplicon size is expected to be 88 bp (Table 1). The size of the TwistAmp exo probe is 52 bases long (Table 1), and the first 28 bases at the 5′ end of the probe overlap with the RPA ZV-R primer. The 32^nd^ base at the 5′ end is a thymine, which was replaced with a dT-FAM (fluorescein amidite), the 33^rd^ base at the 5′ end is an adenine, which was replaced with a THF (tetrahydrofuran), the 34^th^ base at the 5′ end is a thymine, which was replaced with a dT-BHQ1 (Black Hole Quencher 1). This design meets the criteria that there are at least 30 bases 5′ of the THF, and at least 15 bases 3′ of the THF^25^. It also meets the criteria that there are fewer than 5 bases between the fluorophore (FAM) and the quencher (BHQ1), and there is only a single THF between FAM and BHQ1. A Spacer C3 was added to the 3′ end of the probe to block extension.

Real-time RT-RPA reactions were set up using the TwistAmp exo RT kit according to manufacturer’s instructions (TwistDx, Cambridge, United Kingdom). Optically clear 0.2 mL low-profile PCR strips with flat caps were used in this assay. For each 12.5 μL volume reaction, 420 nM each of RPA ZV-F and RPA ZV-R primers, 120 nM of the TwistAmp exo probe, and 2 μL of the RNA template were mixed and centrifuged briefly before adding 14 mM of magnesium acetate to start the reaction.

In this report, RT-RPA was first performed using fluorescence detection in the FAM channel (excitation 470 nm and detection 520 nm) in an ESEQuant Tubescanner (Qiagen Lake Constance GmbH, Stockach, Germany) with a program maintaining 40 °C for 15 min, with a 30 sec measurement interval. Per instructions from the TwistDx’s protocol, the measurement was paused at 4.5 min to allow reaction tubes being taken out for brief agitation and then placed back in the tube scanner to resume fluorescence measurements immediately. The Threshold time (Tt) of isothermal amplification reaction was determined by using the first derivative of the fluorescent signal curve. When the first derivative first becomes greater than or equal to 0.1, that time point is considered as the Tt of that assay. Instead of using 50 μL in each RT-RPA reaction as suggested by the manufacturer, only 12.5 μL was used to reduce cost. In addition, a smaller reaction volume (e.g., 5 μL) was found to eliminate the need for agitation in order to achieve the sensitivity claimed by the manufacturer^30^.

### Low-cost real-time RT-RPA assay using the 3D printer

Although the ESEQuant Tubescanner is a small portable device that can collect fluorescent signal changes over time for up to 12 tubes, it is also an expensive device (over $7,000 per detector). Therefore, the possibility of using a cellphone as an alternative tool to collect fluorescent signals was explored. Real-time RT-RPA was performed on the 3D printer-processed ZIKV template using the heated bed or the extruder of the 3D printer as the heat source to incubate the master mix. To monitor the green fluorescence from Fluorescein dye, we first tried a few blue LED or UV-LED flashlights as an excitation light source, but the results were not satisfactory. We therefore built our own LED array (a row of 8 LEDs 9 mm apart) using individually packaged blue LEDs (470 nm, 5-mm in diameter, 08R2921, Newark.com) as our excitation light source. This particular LED model provides high intensity (4.1 cd luminous intensity) and wide viewing angle (30°). The array was powered by a 9 V battery. To capture the green fluorescence emitted from the blue LED illuminated reaction tubes, a cell phone (Samsung Note 3) with plastic orange filter taken from a gel viewing box (Mini LED Transilluminator, I/O Rodeo, Inc. Pasadena, CA) placed over the camera lens were used to take photos of the tubes every 30 seconds with a $3.95 Android camera app with locked-focus, locked-exposure, and time-lapse photography functions (Camera FV-5 installed on a Samsung Note 3 smartphone). The time-lapsed photos were analyzed visually to identify when the signal intensity of the tubes was greater than the ZIKV-negative urine sample control or NTC reaction signals. We also viewed the fluorescence of the tubes using a blue LED gel viewing box (Lonza, Walkersville, MD). The emission was viewed and recorded using the same orange filter and camera.

### RT-PCR using Thermos Thermal Cycler

After ZIKV RNA templates extracted by the 3D printer were first amplified by real-time RT-PCR using a commercial thermal cycler, the same templates were amplified by RT-PCR using the TTC^40^,^41^. The TTC is a practical solution for bringing a low-cost, simple-to-operate, and rapid thermal cycler to underserved and developing populations. We previously demonstrated rapid PCR and RT-PCR thermal cycling with the TTC at a rate of 15 to 30 sec per cycle^40^,^41^. The TTC uses a very simple design that performs PCR amplification based on the “archaic” method of “hand-transferring” reaction tubes through a series of water baths, minimizing the temperature ramping time needed for PCR tubes to reach thermal equilibrium. The automation of the PCR reactions is controlled by an Arduino microcontroller and the actions of servomotors. After the RT-PCR is completed, the fluorescent signal from these reaction tubes can be viewed using the same LEDs and smartphone setup as described earlier. Each TTC costs less than $200 to build.

With the TTC, numerous reactions were performed in glass capillary tubes. The protocol consisted of 5 min of reverse transcription, followed by 10 sec of RT inhibition, and then 40 cycles of 5 sec of denaturation and 30 sec of annealing/extension, abbreviated as 300 s/10 s/40 × (5 s/30 s). The shortest protocol tried that gave a reasonably good performance of target amplification was 30 s/10 s/40 × (5 s/10 s). It should be noted that when glass capillary tubes were used, the master mix prepared using SuperScript III Platinum One-Step Quantitative RT-PCR system (Life Technologies) did not produce amplicons, likely caused by the adsorption of reverse transcriptase and polymerase onto the glass tube wall, which effectively reduced the concentration of the enzymes for the production of cDNA and amplicons. Therefore, when glass tubes were used in the TTC, the master mix was prepared using the One Step PrimeScript RT-PCR Kit (Takara/Clontech, Mountain View, CA), which was compatible with glass tube PCR reactions. To provide a control for comparison of amplification efficiency, a shorter commercial protocol was also performed. This 30 s/10 s/40 × (5 s/10 s) commercial thermal cycler reaction took ~35 min to complete.

### Gel electrophoresis analysis

Amplicons from RT-PCR were evaluated using a 2.2% pre-casted gel (Flash Gel by Lonza, Walkersville, MD) at 275 V for 7 min (4 μL mix added to 1 μL of loading dye). The resulting gel images were captured using a cell phone camera. The typical ladder sizes used in the gel were 50, 100, 150, 200, 300, 500, 800, and 1500 bp.

### Image analysis

Fluorescent intensity of images captured by cell phone camera was quantitated by ImageJ software developed by NIH^42^. Mean gray values of the fluorescent areas selected were reported.

### Statistical analysis

Differences between the 3D printer method and the Qiagen spin-column method were examined. Quantification Cycle (Cq) values from both methods were analyzed using paired t-test with an online software (QuickCalcs of GraphPad, La Jolla, CA). A *P*-value less than 0.05 was considered statistically significant. The Cq values were also analyzed by the Levene’s test for equality of variances^43^.

## Results

### Performance of 3D printer-based RNA extraction tested by real-time RT-PCR

**Figure 2:**
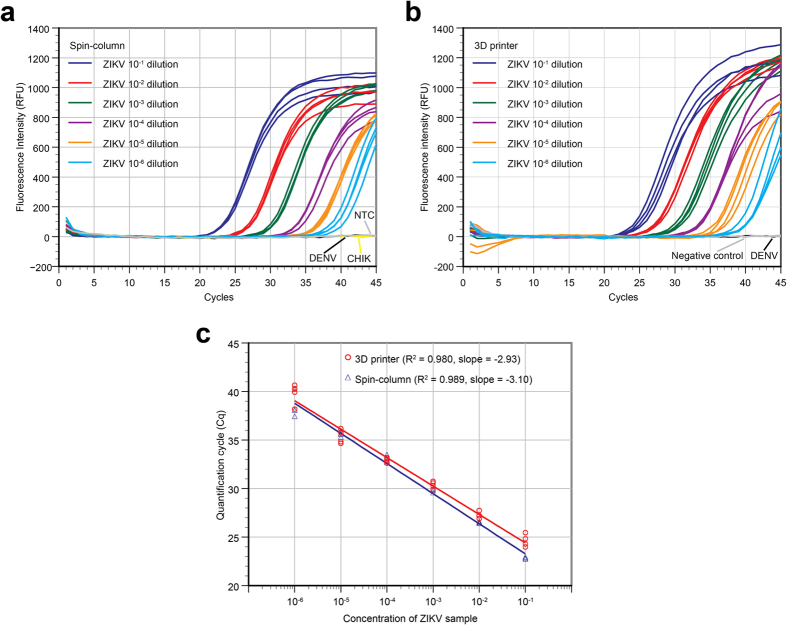
Real-time RT-PCR data from serially diluted ZIKV samples spiked in human urine. (**a**) RNA templates were extracted from each concentration 4 times, using the spin-column protocol. Dengue viral RNA (DENV) and chikungunya viral RNA (CHIK) were used as negative controls of this real-time RT-PCR assay. NTC was the no-template control reaction. (**b**) RNA templates were extracted from each concentration 4 times, using the 3D printer protocol. Urine sample without ZIKV (Negative control), and dengue virus samples (DENV) were used as negative controls in the extraction process. (**c**) Standard curves were plotted from both the spin-column protocol and 3D printer protocol data. The R^2^ value and slope of the plot are included and the Qiagen spin-column extraction method appears to be slightly better with samples of higher concentrations (10^−1^ and 10^−2^ dilutions). Nevertheless, the results show that ZIKV concentrations as low as 10^−6^ can be effectively extracted by both methods and detected by a commercial thermal cycler using RT-PCR.

**Figure 3:**
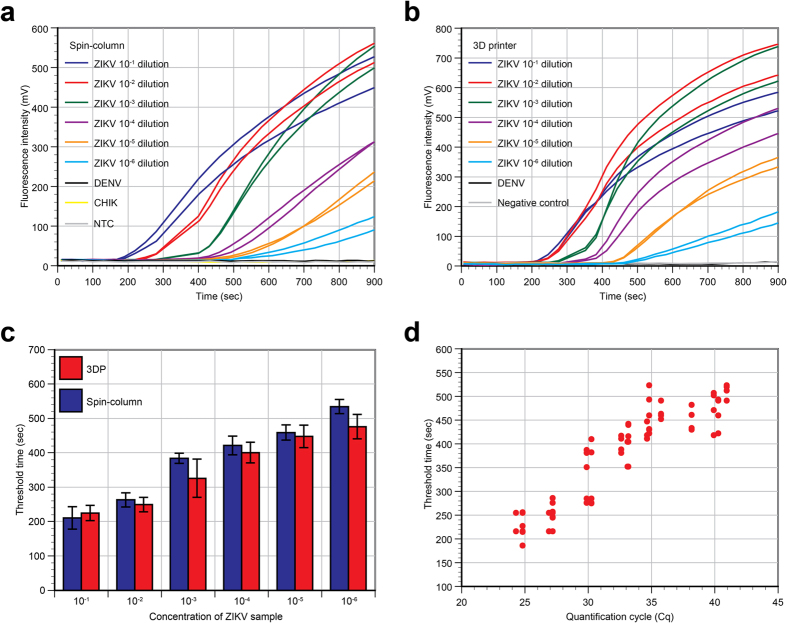
Real-time RT-RPA performed in the ESEQuant Tubescanner. (**a**) Representative real-time RT-RPA data of spin-column processed samples (n = 2) from serially diluted ZIKV samples spiked in urine. Dengue viral RNA (DENV) and chikungunya viral RNA (CHIK) were used as negative controls. NTC was the no-template control reaction. (**b**) Representative real-time RT-RPA data of 3D printer processed serially diluted ZIKV samples spiked in urine (n = 2). Urine sample without ZIKV (Negative control), and dengue virus samples (DENV) were used as negative controls in the extraction process. (**c**) Threshold time (Tt) vs concentration of the ZIKV samples processed by the spin-column method or the 3D printer method. Results show that as low as 10^−5^ diluted ZIKV sample can be extracted by the 3D printer method and reliably detected by RT-RPA. (**d**) Comparison between real-time RT-PCR (X-axis) and RT-RPA (Y-axis) for the detection of ZIKV. The RT-RPA reaction was much faster than the real-time RT-PCR even for samples with large Tt values. For example, the 10^−5^ dilution sample needed a Cq of ~35 cycles in RT-PCR (at 46.7 min of the entire 57 min protocol) to get a positive signal over the background; when using RT-RPA, less than 500 sec was needed.

The performance of the 3D printer extraction on simulated urine samples was compared to the gold-standard spin-column based extraction method (Qiagen) by performing nucleic acid extractions of 10-fold serial dilutions of ZIKV-positive samples (10^−1^ to 10^−6^). Quadruplicate samples of each dilution were extracted using both methods, and Cq values from the real-time RT-PCR of the templates were analyzed. The real-time RT-PCR curves of the two sets of templates were plotted separately. The plots show that RT-PCR amplified ZIKV RNA down to 10^−6^ dilution (5.0 PFU/mL or 0.5 PFU/extraction with 100 μL sample input) from both spin-column (Fig. 2a) and 3D printer processed (Fig. 2b) samples. The ZIKV RT-PCR assay is also not reactive towards the dengue and chikungunya RNA as well as the negative urine samples.

The plots of Cq vs cycle number from both sets of templates are shown in Fig. 2c. The plots suggest that the spin-column method produced a slightly higher concentration of template as smaller Cq values were consistently obtained. The R^2^ values and slope of the plot are listed in Fig. 2c. Cq values between the two methods were within 2 cycles in most cases. The R^2^ value (0.980) of the plot using the 3D printer indicates that extraction efficiency was linear over 6 orders of magnitude of the concentrations tested. Amplification efficiency of samples prepared by the 3D printer was found to be 119% (slope of −2.93, efficiency = 10^(−1/slope)^ −1), which indicates that the automated process provided an excellent yield of nucleic acids with minimal PCR inhibitors. The paired *t*-test result indicates that the 3D printer-processed samples had a statistically higher Cq values at 10^−1^ and 10^−2^ dilutions (*P* values of 0.009 and 0.015) compared to the spin-column method. However, for 10^−3^ to 10^−6^ dilution samples, *P* values were 0.081, 0.941, 0.572 and 0.166 respectively; these results indicate no statistical significant differences were observed between the Cq values using these two methods with lower concentrated samples. This difference in efficiency could be due to the use of carrier RNA in the spin-column protocol, which makes the binding of RNA onto the silica membrane more effective. While not shown, the performance of the automated RNA extraction by the 3D printer is identical to those performed manually by following the manufacturer’s suggested protocol. Therefore, the adaptation of the manual MP-based protocol to the automated 3D printer approach does not negatively impact the yield. Additionally, the 3D printer platform allows the use of different formats of microplates or cartridges. It is feasible to use more than 1 mL of urine sample per extraction (data not shown); while the standard spin column approach can only handle a small sample input (200-μL) unless the user manually splits and then loads the lysate onto the spin column successively. The ability to use a larger sample volume can effectively increase the overall assay sensitivity.

Figure 2 appears to show that the 3D printer-based extraction method exhibited larger performance variability than that of the spin-column method. Therefore, we performed the Levene’s test to analyze the variances of Cq values from both extraction methods. For the 10^−1^ and 10^−5^ dilution samples (*P* values of 0.023 and 0.008), the 3D printer method indeed exhibited a larger variance of Cq values than that of the spin-column method. However, for the 10^−2^, 10^−3^, 10^−4^ and 10^−6^ dilution samples, there is no significant difference in variance of Cq values between these two methods (*P* values of 0.159, 0.092, 0.421 and 0.834 respectively). Therefore, the 3D printer method showed only a slightly lower consistency in RNA extraction performance than the spin-column method. It is not unexpected as the spin-column method is a fully developed product while our platform is in its early stages of development and limited testing and replicates have been performed.

While the 3D printer method produced slightly inferior Cq values for the same sample, it did not lead to a less sensitive real-time RT-PCR assay using the commercial thermal cycler (both at 10^−6^ dilution samples). Additionally, the 3D printer method utilized a much shorter time to complete the same number of samples (14 min vs 45 min by the spin-column method). Furthermore, the spin-column method needs extensive user intervention between each wash while the 3D printer approach is hands-free after the process starts. Therefore, the user can use the waiting time during extraction to prepare for the amplification assay. Overall, the 3D printer offers a faster and more user-friendly extraction protocol while delivering the same dynamic range and sensitivity in a RT-PCR assay.

### Performance of 3D printer-based RNA extraction tested by real-time RT-RPA

After the RNA templates were tested by real-time RT-PCR, RT-RPA was used to check if the primers and exo-probe provided faster and more sensitive amplification and detection of ZIKV RNA via RT-RPA using the ESEQuant Tubescanner. The plots of fluorescent signal over time using the spin-column- and 3D printer-processed templates are shown in Fig. 3a and b, respectively. A quick look at the plots shows that, similar to the RT-PCR experiment, RT-RPA on the 3D printer- and spin-column-processed RNA offered roughly the same concentration range (10^−1^ to 10^−6^) when amplification took place in the ESEQuant Tubescanner. At minimum, the data indicated that a qualitative “Yes or No” test can be performed using real-time RT-RPA with the Tubescanner instrument.

The Tt of real-time RT-RPA reactions at each dilution was plotted and shown in Fig. 3c. Real-time RT-RPA results with RNA extracted by the both methods shows that the 10^−1^ and 10^−2^ ZIKV samples had Tt of under 5 min. It should be noted that the final fluorescent signal from reactions with extremely high template concentration can occasionally be attenuated. Similar results have been observed by others^44^,^45^. It is believed that a high nucleic acid concentration can inhibit the RPA reaction since proteins within the RPA reactions will bind to the DNA or RNA template; less protein is then available to bind to the primers for amplification if an excess amount of template is already in the reaction.

For the real-time RT-RPA reaction with RNA extracted from the 10^−3^ diluted ZIKV sample, the Tt value was under 400 s (6.7 min). For the 10^−4^ diluted ZIKV sample reaction, the Tt value was under 450 s (7.5 min). For the 10^−5^ and 10^−6^ diluted ZIKV sample reactions, the Tt values were approximately between 450 and 600 s (10 min). The average Tt between 10^−5^ and 10^−6^ diluted ZIKV samples is not very different, and there is wider variation in the Tt when the 10^−6^ samples were tested. This suggests that the RT-RPA assay’s limit of detection is likely between these two dilutions. The signal from the negative controls remained low after 10 min of incubation. The RT-RPA primers and probe used are also confirmed to be specific towards ZIKV sequences as the use of DENV-1-4 RNA template did not lead to a false positive result.

Figure 3d is a comparison between real-time RT-PCR (X-axis) and RT-RPA (Y-axis) for the detection of ZIKV. It shows that the RT-RPA reaction is much faster than the real-time RT-PCR, even with samples of high template concentrations (e.g., Cq ~22). The shortest RT-PCR protocol run on a thermal cycler took 20 and 36.5 min to complete 20 and 40 cycles, respectively. In contrast, most RT-RPA results can be determined in less than 10 min. It should be noted that RT-RPA is a relatively new method and is considered a semi-quantitative method. The addition of fluorescently labeled probes to enable real-time RT-RPA will not turn the method into a quantitative one, although it does have the ability to differentiate samples with significantly different concentrations of RNA (1 order). Also, data (Fig. 3c and d) showed that there are relatively high variations in Tt among the RT-RPA reactions with the same concentration of template. We suspect this is partly due to the use of clouding agent in the reaction and that subsequently, the manufacturer instructs users to agitate the reaction mix a few minutes after the reaction has started. The extent and consistency of agitation can be difficult to maintain between runs or users so the variation is expected to be slightly higher than in traditional PCR reactions.

When compared to the real-time RT-PCR assay (Fig. 2), which can definitively detect templates from 10^−6^ diluted samples, the lowest template concentration reliably detected by real-time RT-RPA in the ESEQuant Tubescanner was likely between the 10^−5^ and 10^−6^ dilution, given the similar Tt values. This indicates that the real-time RT-RPA assay is slightly less sensitive than traditional real-time RT-PCR methods. This is not unexpected; it was reported that analytical sensitivity of a RT-RPA assay developed for Ebola was one order less sensitive than RT-PCR when tested with an RNA standard^46^. Because the primers and probes sequences and concentrations that were initially designed can be further modified, it is very likely that this new assay can become more sensitive when optimized.

### Amplification of ZIKV RNA by real-time RT-RPA using the 3D printer and smartphone

**Figure 4:**
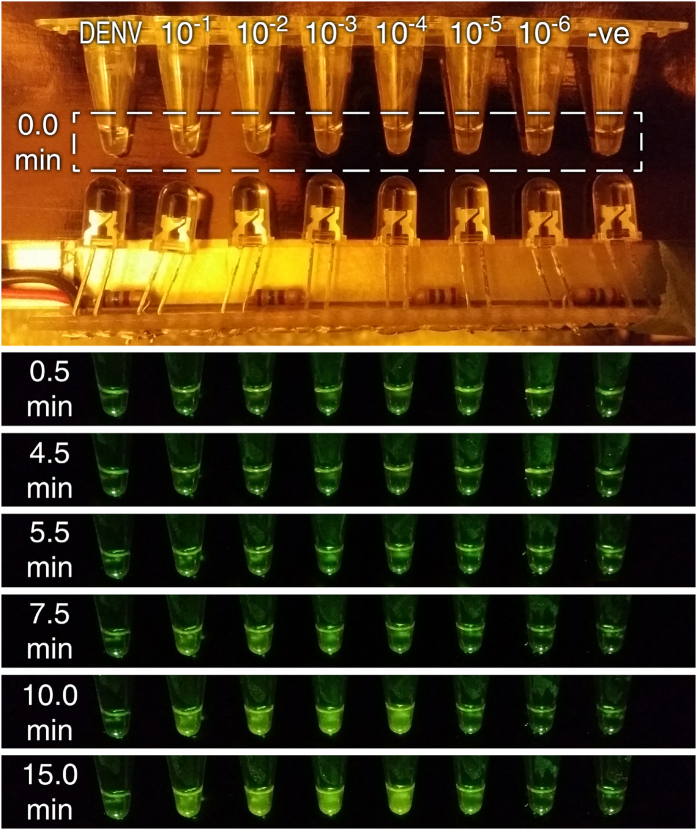
Selected time-lapse photos of the reaction tubes with RT-RPA reactions performed on the 3D printer’s heated bed. The top photo was taken with ambient light. It shows the placement of the LEDs and reaction tubes. The photos show that one can identify most of the ZIKV positive samples within 10 min (the tubes were vortexed briefly after 4.5 min). One can determine that the samples (10^−1^, 10^−2^, 10^−3^, 10^−4^, and 10^−5^ dilution) can be detected by using phone camera as early as 5.5, 5.5, 6, 7.5, and 12 min, respectively. The fluorescent signal growth at the 10^−6^ tube could not be confidently confirmed. Signals of both negatives remained low throughout the 15 min incubation. Extending the imaging time from 10 to 15 min did not further increase the sensitivity although the final fluorescent signals are higher in the ZIKV positive tubes.

While RNA extraction is critical in the work flow of rapid, reliable, and consistent molecular diagnostics, it is equally important to have a rapid, low-cost, and accurate method to detect the extracted RNA. After demonstrating that RT-RPA of the 3D printer-extracted ZIKV RNA can be amplified and detected using the ESEQuant Tubescanner, the next step in the process was to demonstrate the low-cost and rapid detection of ZIKV from simulated samples using the 3D printer’s heating element (heated bed and the extruder). A smartphone camera was used to record fluorescent signal change over time during the RT-RPA reactions. Figure 4 shows a series of time-lapse photos of the RT-RPA as taken by the cellphone (0, 0.5, 4.5, 5.5, 7.5, 10, and 15 min). The 8 samples in a single strip contained 3D printer extracted templates from 10^−1^, 10^−2^, 10^−3^, 10^−4^, 10^−5^ and 10^−6^ diluted ZIKV samples in urine. The two controls at both ends of the strip were templates from DENV-1-4 RNA (left) and negative control (ZIKV-negative human urine) (right). Blue LEDs with excitation wavelength of 470 nm illuminated the reaction tubes. An animated gif file showing the changes in fluorescent signal in each tube was included as Supplementary Animation S1.

Similar to the ESEQuant Tubescanner-derived result, the photos show that the sample containing ZIKV can be identified in as little as 5.5 min with the 10^−1^ and 10^−2^ samples. By inspecting the time-lapsed photos, one can determine with unaided eye within 12 min (6.0, 7.5, and 12.0 min, respectively) that the 10^−3^, 10^−4^, and 10^−5^ samples all showed noticeably higher signal than the controls. Using ImageJ, the photos were analyzed and the signal growth in each sample tube was plotted for a period of 15 min (Supplementary Fig. S1). This figure suggests that ZIKV detection from 10^−1^ to 10^−5^ dilution samples can be confirmed by photographs. Using the time-lapse function of a smartphone, the level of template concentration can be identified based on fluorescent signal change over time. The Tt value derived from the photos is higher, but it is not too far from the values collected using the much more expensive Tubescanner device (Fig. 3c). Repeated RT-RPA runs indicated that RT-RPA can be amplified and detected consistently in these 4 orders (10^−1^ to 10^−4^) of concentrations in less than 10 min. The 10^−5^ diluted samples showed higher signal than the negative control between 10 and 12 min in most cases.

Similar to the RT-RPA performed by the ESEQuant Tubescanner, this current setup was not able to reliably detect ZIKV in the 10^−6^ diluted samples. Overall, this low-cost approach of using smartphone and heated bed of the 3D printer essentially achieved the same sensitivity as the more expensive real-time RT-RPA setup using the ESEQuant Tubescanner, confirming the feasibility of monitoring real-time RT-RPA signal growth using LEDs and a smartphone camera. Supplementary Fig. S2 shows another set up for real-time monitoring of RT-RPA reaction for ZIKV. The LEDs are mounted 9-mm apart on a holder and placed above the cap of the tubes to illuminate the reaction tubes above. The 4 tubes contained templates from negative urine, 10^−4^, 10^−3^, 10^−2^ diluted ZIKV samples. It is clear from the photos that at 6 min, the 10^−2^ diluted sample started to show a noticeable increase in fluorescence.

In addition to the heated bed, the extruder in a 3D printer can also be used to incubate tubes for RPA. The same templates in the last section were amplified on an aluminum block that was heated by the extruder. Due to the small sizes, the temperature of the extruder was set at 75 °C in order to raise the temperature of the much larger aluminum block to 40 °C. The same LEDs and camera set up was used to take time-lapse photos of the reactions, and the same concentration (10^−1^ to 10^−5^ dilution) range of templates was detected. A gif file showing the changes in fluorescent signal in each tube was included as Supplementary Animation S2. The ability to use the extruder to provide heat means a lower cost 3D printer without a heated bed can be used to perform extraction and RT-RPA amplification.

If one PFU is equivalent to 1,000 genome copies of RNA, the 10^−5^ diluted ZIKV sample (50 PFU/mL or 5 PFU per extraction) would have had 5,000 copies of RNA in urine per extraction or 100 copies of RNA in 2 μL of template used in each RT-RPA reaction (assuming 100% capture and release of RNA by MPs). Because the viral load found in human infections ranges from 10^2^ to 10^6 ^PFU/mL^10^,^11^,^12^,^13^,^14^ and the recent paper by Gourinat *et al*. shows evidence of virus secretion in urine (0.7–2.2 × 10^6^ RNA copies/mL)^18^, these data suggest that clinically relevant RNA concentrations can be detected in less than 12 min using real-time RT-RPA assay and results display high specificity. A recent report also shows that RPA reagents were able to detect 10 HIV-1 DNA copies after storage at 25 and 45 °C for up to 3 and 12 weeks, respectively^30^. Together, there is a high probability that this low-cost approach and the use of RT-RPA can be implemented in low-resource settings without the need of a cold chain.

### Rapid amplification and detection of ZIKV by TTC

Multiple studies have verified the detection of ZIKV by the gold-standard RT-PCR approach; however, this investigation demonstrated the capability to provide rapid and accurate ZIKV diagnostics using RT-PCR in a lower cost approach. To this end, the recently developed TTC was used to amplify the 3D printer-processed ZIKV RNA template by RT-PCR and post-amplification fluorescent detection was used to identify ZIKV-positive samples. Using the TTC, 40 cycles of RT-PCR with the 30 s/10 s/40 × (5 s/10 s) protocol was completed in 11 min and 35 sec. In comparison, the commercial thermal cycler needed 35 min to complete the same protocol. The post-PCR fluorescent intensity of the glass capillary tubes was aptly captured using a smartphone camera.

**Figure 5:**
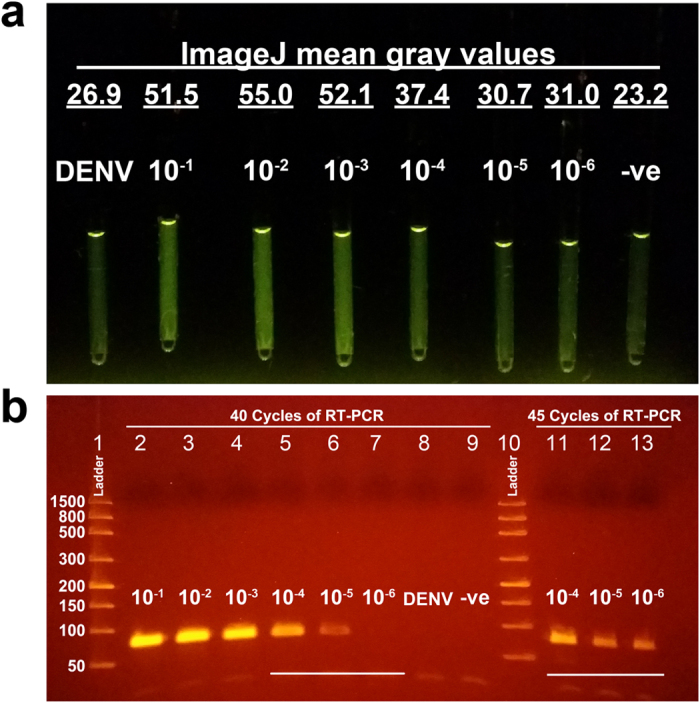
Rapid RT-PCR of ZIKV RNA by the TTC. RT-PCR of template RNA extracted from different concentrations of ZIKV (10^−1^ to 10^−6^ dilutions) was performed by the TTC using glass capillary tubes. Negative controls were templates from ZIKV-negative urine and dengue viral RNA. The 40-cycle reaction was completed in 11 min and 35 sec. (**a**) Image showing the fluorescent intensity of the glass capillary tubes as illuminated by blue LEDs. Samples as low as 10^−4^ can be seen as positive when compared to the negative controls. Endpoint fluorescent intensity was quantitated by ImageJ, and underlined values are mean gray values of each reaction tube. (**b**) A gel electrophoresis image showing the gel bands of the corresponding samples shown in panel a (lanes 2 to 9). While not identified as ZIKV-positive by the photo in panel a, the 10^−5^ sample shows a faint band. Lanes 11 to 13 are gel bands of amplicons from the 10^−4^ to 10^−6^ dilution templates that have undergone 45 cycles. Even the template from the 10^−6^ dilution sample produced a noticeable gel band. This shows that RT-PCR by the TTC can be as effective as a commercial real-time PCR system while being much faster.

The photo in Fig. 5a shows higher fluorescence intensity from tubes with 10^−1^, 10^−2^, 10^−3^, and 10^−4^ diluted samples. Fluorescence intensity from samples with lower concentrations (10^−5^ and 10^−6^ diluted ZIKV samples) was not distinguishable from the two negative controls in the figure (template from ZIKV negative urine and dengue RNA template). To confirm specificity and correct product size, gel electrophoresis was performed (Fig. 5b, lanes 2 to 9). The 10^−5^ diluted sample, while not distinguishable in the photo of the glass tubes, gave a positive result albeit in the form of a faint band. The negative controls of dengue RNA template and ZIKV-negative urine did not produce visible amplicon bands. RT-PCR performed by the TTC is slightly less sensitive than when performed by a commercial cycler (10^−6^ dilution samples were not detectable by the TTC). It is possible that the difference is due to the fact that 12 μL reaction was used per glass tube while the commercial reactions had 20 μL per plastic tube. Therefore, the absolute number of templates in each reaction available for amplification is 40% smaller when the glass tube is used. The overall amplification performance can be negatively affected when the concentration of template reaches the stochastic territory. Also, although the Takara master mix is compatible with amplification within a glass container, adsorption of polymerases to the untreated glass wall (although to a lesser extent) is still possible, and this too can reduce amplification efficiency. To improve per-cycle efficiency, users can use longer incubation time in each cycle or add additional PCR cycles in the run.

Since the TTC did not generate enough amplicons in 40 cycles for fluorescent imaging at concentrations below 10^−5^, a higher number of RT-PCR cycles could be used to improve the sensitivity. Therefore, 45 cycles of PCR were performed by the TTC on 10^−4^, 10^−5^, and 10^−6^ diluted samples and the increased number of cycles resulted in positive results as confirmed by gel electrophoresis (Fig. 5b, lanes 11 to 13). The 5 extra cycles only took an additional 1 min and 22 s to complete, bringing a 45 cycle reaction’s run time to 12 min 57 s. Therefore, the additional cycles compensated for the slightly suboptimal per-cycle PCR efficiency, and the overall sensitivity of the RT-PCR assay performed by the TTC was as high as that provided by commercial units. The clear advantage of using a TTC is that the reactions can be accomplished in under 13 min with only one minute of post-amplification imaging needed to view the tubes by fluorescence and record the signal by a smartphone. This is a time savings of 21 min over the 40-cycle RT-PCR commercial run. In short, the speed of RT-PCR by TTC also enables the analysis of ZIKV samples in less than 30 min.

## Discussion

A very low-cost but high performing molecular approach for ZIKV detection has now completed its initial proof-of-concept demonstration. Using a modified, entry-level 3D printer to perform automated MP-based RNA extraction and purification, 8 to 12 ZIKV spiked urine samples can be processed in less than 15 min. The 3D printer-operated extraction can handle samples with a 6-log difference in target concentration. The efficiency of the outlined extraction process without the use of carrier-RNA is only slightly inferior to the gold standard spin-column; however, it is sufficient for the detection of ZIKV. Subsequent amplification and detection of the ZIKV RNA template by real-time RT-RPA and RT-PCR with post amplification detection can be done in 12 and 15 min, respectively. Testing of multiple samples for ZIKV in 30 min with minimal hands-on time has been accomplished. Clinically relevant sensitivity (5 PFU/mL at the 10^−6^ dilution) was attained using our current approach; and cross-reactivity with dengue and chikungunya RNA template was not observed, thereby demonstrating sufficient sensitivity and specificity for ZIKV detection. The speed and sensitivity of the system, while sufficient and an improvement on current capacity, could be further optimized. With its initial proof-of concept successfully demonstrated, the system reported here potentially can offer the most immediate impact in the diagnosis of ZIKV by offering a rapid, easy-to-operate, and low-cost approach without compromising the sensitivity and specificity needed to tackle this global health crisis.

## Additional Information

**How to cite this article**: Chan, K. *et al*. Rapid, Affordable and Portable Medium-Throughput Molecular Device for Zika Virus. *Sci. Rep.* **6**, 38223; doi: 10.1038/srep38223 (2016).

**Publisher's note:** Springer Nature remains neutral with regard to jurisdictional claims in published maps and institutional affiliations.

## Supplementary Material

Supplementary Animation S1

Supplementary Animation S2

Supplementary Information

## Figures and Tables

**Table 1:** Sequence of primers and probes used in this study.

PCR primers/Probes	Sequence	Target	Amplicon Size	Reference
ZIKV 1086	5′ CCG CTG CCC AAC ACA AG 3′	E (envelope gene)	77 bp	10
ZIKV 1162c	5′ CCA CTA ACG TTC TTT TGC AGA CAT 3′			
ZIKV 1107-FAM probe	5′ FAM- AGC CTA CCT TGA CAA GCA GTC AGA CAC TCA A-BHQ-1 3′			
RPA ZV-F	5′ CAT ATA CTT GGT CAT GAT ACT GCT GAT TGC C 3′	prM (premembrane gene)	88 bp	This study
RPA ZV-R	5′ CAT ACC TTC CAC AAA GTC CCT ATT GCT GAC 3′			
RPA exoZV-FAM probe	5′ TAC CTT CCA CAA AGT CCC TAT TGC TGA CTC C-T (FAM)-dSpacer-T(BHQ-1)-GC ACC TGA TGC TGT ATG C-Spacer C3 3′			
