# Supplementary figures and images for: Rapid, Affordable and Portable Medium-Throughput Molecular Device for Zika Virus
Source: Sci Rep. 2016 Dec 9;6:38223. doi: 10.1038/srep38223 (PMC5146750; doi:10.1038/srep38223)

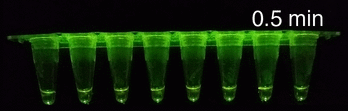

Supplement: Supplementary Animation S1 [file srep38223-s1.gif]

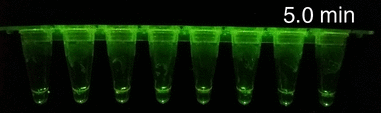

Supplement: Supplementary Animation S2 [file srep38223-s2.gif]
