# Supplementary material for: Rapid, Affordable and Portable Medium-Throughput Molecular Device for Zika Virus
Source: Sci Rep. 2016 Dec 9;6:38223. doi: 10.1038/srep38223 (PMC5146750; doi:10.1038/srep38223)
Supplement: Supplementary Information [file srep38223-s3.pdf]

Rapid, Affordable and Portable Medium-Throughput Molecular Device for Zika Virus

Kamfai Chan[1], Scott C. Weaver[2], Pui-Yan Wong[1], Sherly Lie[1], Eryu Wang[2], Mathilde Guerbois[2], Siva Praneeth Vayugundla[1], and Season Wong[1,*]

[1] AI Biosciences, Inc., College Station, TX 77845, USA.

[2] Institute for Human Infections and Immunity and Departments of Microbiology & Immunology and Pathology, University of Texas Medical Branch, Galveston, TX 77555, USA.

*** Corresponding author**

Season Wong

AI Biosciences, Inc.

1902 Pinon Dr., Suite C

College Station, TX 77845-5816

Phone: 1 979 268 1091

Email: season.wong@aibiosciences.com

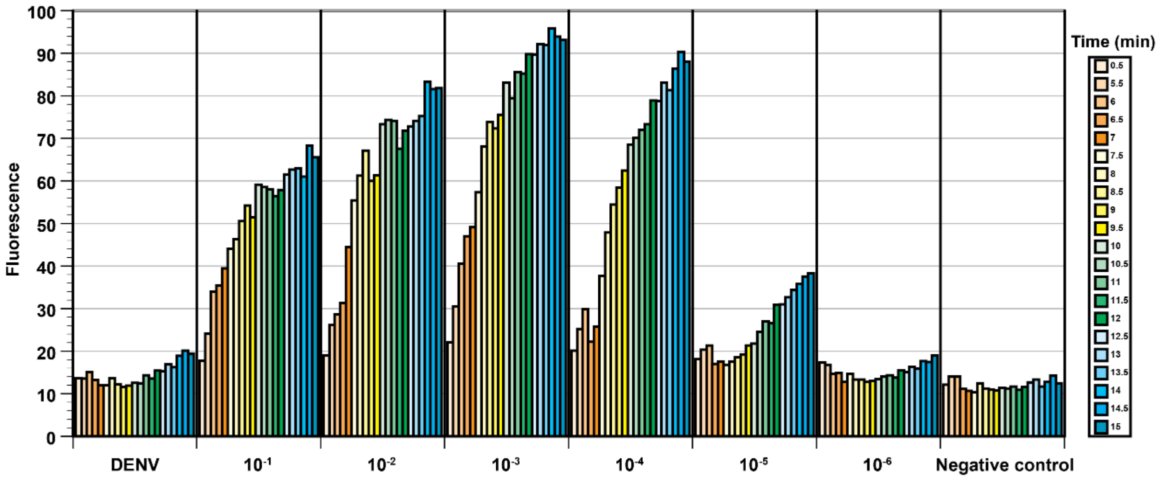

Supplementary Figure S1. Fluorescent intensity of images of 3D printer-based real-time RT-RPA quantitated by ImageJ. Individual image of real-time RT-RPA reactions captured by smartphone camera at time 0.5 min and 30 sec interval from 5.5 min to 15 min. Fluorescent intensity was quantitated by ImageJ software in mean gray values.

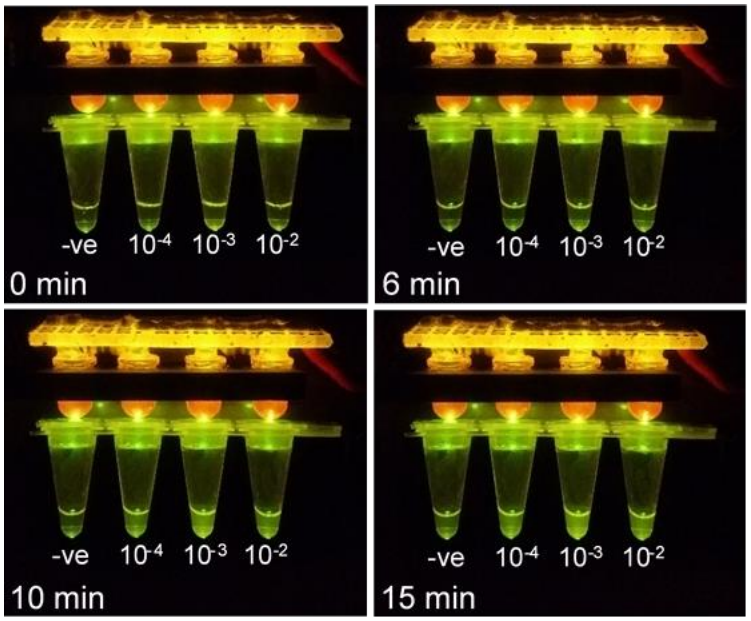

Supplementary Figure S2. Time-lapse photos of the RT-RPA reaction tubes performed on the 3D printer's heated bed. Unlike **Fig. 4** in which LEDs were placed at the bottom of the PCR tubes, LEDs shown here were placed above the cap of the tubes and illuminated the reagent mix downwards. The templates from left to right are dengue RNA (negative control), and ZIKV RNA 10^{-4}, 10^{-3}, and 10^{-2} dilutions. The photos show that one can identify the sample containing ZIKV in as little as 6 min with the 10^{-2} samples. By 10 min, one can identify all three samples as positive when compared to the negative control. Additional 5 min incubation did not alter the result. The 10^{-2} sample's final fluorescence was lower than the 10^{-3} probably due to excessive template that interacted with the single stranded DNA binding proteins, thus lowering their amount for the RPA reactions.

Supplementary Animation S1. Animated gif file of the time-lapsed photos from RT-RPA reactions performed on a heated bed of the 3D printer. Samples from left to right: dengue RNA, 10^{-1} to 10^{-6} diluted ZIKV sample in urine, negative urine.

Supplementary Animation S2. Animated gif file of the time-lapsed photos from RT-RPA reactions performed on a aluminum surface heated by the extruder heater. Samples from left to right: dengue RNA, 10^{-1} to 10^{-6} diluted ZIKV sample in urine, negative urine.
